# Scrolling for support: informational, emotional, and social support in adult ADHD Facebook groups

**DOI:** 10.3389/fpsyt.2025.1755437

**Published:** 2026-01-26

**Authors:** Michal Isser, Tali Gazit

**Affiliations:** Department of Information Science and Applied Artificial Intelligence (AI), Bar-Ilan University, Ramat Gan, Israel

**Keywords:** ADHD, Facebook, social media, support, well-being, online support groups

## Abstract

**Introduction:**

This study examines Facebook-based ADHD support groups for adults, addressing a gap in the understanding of how participation in ADHD-specific online communities contributes to informational, emotional, and social support, and how these processes relate to mental well-being.

**Methods:**

An inductive qualitative design was employed, based on semi-structured interviews with twenty adults (nine men and eleven women) diagnosed with ADHD who actively participate in large Facebook support groups comprising tens of thousands of members. The interviews were analyzed inductively to identify recurring themes related to engagement and support within these online communities.

**Results:**

The analysis revealed that Facebook-based ADHD groups function as supportive networks that provide informational, emotional, and social support. Participants described these groups as safe spaces for sharing experiences and advice, fostering a sense of belonging, reducing feelings of isolation, and enhancing engagement within the community.

**Discussion:**

The findings underscore the potential of online peer support as a complementary resource for improving coping, adjustment, and mental well-being among adults with ADHD. These results suggest practical implications for psychoeducation and clinical interventions that incorporate digital social support environments.

## 1 Introduction

Attention Deficit Hyperactivity Disorder (ADHD) is the most diagnosed neurodevelopmental disorder in childhood (1). It is characterized by a persistent pattern of inattention, hyperactivity, and impulsivity that is inconsistent with the individual’s developmental level and age (2). ADHD is a chronic neurodevelopmental disorder linked to a wide range of negative outcomes, including behavioral problems, risk-taking behaviors, and functional impairments across academic, occupational, and social domains (3, 4). While traditionally conceptualized as a psychiatric disorder in the classical sense, a growing body of literature raises critical questions regarding this framework, highlighting ongoing debates about the nature of attentional and behavioral difficulties (5).

Beyond categorical diagnostic frameworks, contemporary research increasingly conceptualizes ADHD as a dimensional condition existing along a continuum of attentional and executive functioning difficulties (6). From this perspective, attentional challenges vary in severity and impact, encompassing individuals who may not meet formal diagnostic criteria but nevertheless experience meaningful functional difficulties. These challenges transcend regional and cultural boundaries; ADHD is recognized globally as a cross-cultural condition that impacts academic performance, physical health, and mental well-being across diverse populations (7, 8).

Recent literature highlights the potential benefits of social media, particularly for individuals with ADHD. In the digital age, social interactions frequently occur through social media platforms, serving various psychological and social functions, such as coping with stress (9), enhancing overall well-being (10), and supporting the development of social identities (11).

Furthermore, it is crucial to examine the interplay between core ADHD symptoms such as impulsivity, executive dysfunction, and hyperfocus and the digital landscape. While text-based, asynchronous platforms like Facebook groups offer the benefit of flexible engagement and time for reflection, they also present unique obstacles regarding information processing and attentional control when compared to synchronous or face-to-face communication. In this context, recent qualitative findings suggest that adults with ADHD often view these online communities as ‘safe spaces’ that bypass the pressures of face-to-face interactions, facilitating more comfortable engagement (12).

Support groups can be especially meaningful for individuals coping with ADHD. Young people with ADHD often experience loneliness, which negatively impacts their self-esteem (13). Additional research indicates that children and adolescents with ADHD report higher levels of loneliness (14), and among individuals with ADHD, loneliness is positively correlated with symptoms of depression, unlike in individuals without ADHD (15).

Despite growing research on ADHD and online support groups, little is known about the lived experiences of individuals participating in ADHD-focused Facebook communities. Understanding these groups is important because they offer a distinctive setting in which people with ADHD seek information, share coping strategies, and engage socially in ways that may shape their well-being and sense of belonging. Prior work has largely focused on parents of children with ADHD (e.g., 16) or on short-term, small-scale focus groups, primarily among young adults (12). The present study addresses this gap by examining sustained participation across a broader age range within large ADHD Facebook groups, adopting an ecological perspective on how these communities operate as ongoing support networks. Specifically, it investigates the emotional, informational, and social support exchanged in these groups, highlighting their role as spaces for sharing experiences, offering advice, and providing mutual encouragement, and thereby revealing the underexplored potential of online platforms to meet the diverse needs of individuals with ADHD.

## 2 Literature review

### 2.1 Social support, well-being and ADHD

Social support can enhance functioning among individuals with ADHD. For example, a supportive partner has been described as helpful in enabling individuals with ADHD to remain organized and manage various life tasks (17). Among young adults with ADHD, the presence of a close friend provided non-judgmental support that facilitated social activities and helped them meet commitments (18). Adults with ADHD have expressed interest in support groups, including online support communities (19). In line with this, recent research shows that adults with self-reported ADHD tend to post more frequently on platforms that provide immediate feedback, reflecting their characteristic difficulty in delaying gratification (20). It is well-established that social support exerts a broad protective influence and can serve as a critical factor in facilitating success for individuals with ADHD across various life domains (21). Reeble et al. (22) highlighted the significance of maintaining a wide-ranging support network for college students with ADHD, indicating that having a larger number of acquaintances may confer greater benefits than relying on a few close friendships. Furthermore, the interaction between ADHD symptom severity and the extent of social support accounted for a substantial portion of the variability in functional impairment. This suggests that students exhibiting higher levels of ADHD symptoms, particularly those with limited support networks, are more likely to experience pronounced functional difficulties.

Beyond functional outcomes, the availability and quality of social support are closely intertwined with the mental well-being of individuals with ADHD. Symptoms of ADHD have been consistently linked to heightened frustration intolerance, diminished self-esteem, reduced achievement motivation, and difficulties in social interaction (23). These ongoing challenges are reflected in lower levels of life satisfaction and well-being compared to individuals without disorder (24, 25). Repeated experiences of failure, rejection, and elevated stress further compromise their mental health (26, 27). In this context, social support functions not only as a buffer against daily stressors but also as a vital contributor to emotional stability, resilience, and overall well-being.

Notably, the findings imply that a greater quantity of more casual or superficial social connections may be more advantageous for this population than a smaller number of deeply intimate relationships. One explanation is that students with ADHD often require extensive support, and distributing support-seeking across multiple individuals may reduce the burden on any single person, thereby creating a more effective overall support system (22). In this regard, Facebook can be conceptualized as a social environment encompassing both strong and weak ties, where users maintain relationships with longstanding friends while simultaneously establishing new connections (28). Thus, social media platforms may serve as valuable resources for individuals with ADHD, providing access to emotional information and social support that can enhance daily functioning and improve well-being. Ginapp et al. (12) found that online communities play a critical role in identity development among young adults with ADHD, in disseminating information about the condition, and in fostering community-building. Their study also revealed that these communities were perceived as positive spaces, allowing participants to accept and come to terms with their diagnosis, and validating their personal experiences.

Importantly, these communities provide both emotional and informational support, helping members share coping strategies, normalize experiences, and strengthen their sense of belonging. However, this topic has been studied only through focus groups (12). To build on existing knowledge, the present study explored the specific dynamics of peer support within ADHD-focused Facebook groups, aiming to understand how online support is expressed across informational, emotional, and social dimensions, and how it contributes to participants’ well-being. As the most prominent social network worldwide with approximately four billion users in the third quarter of 2023 (29) Facebook provides a particularly significant and relevant context for examining these interactions. Accordingly, this study employed a qualitative design using in-depth interviews to capture participants lived experiences and to illuminate the nuanced ways in which support is exchanged within these communities.

### 2.2 Facebook support groups

Online support groups are designed to promote well-being and enhance social interaction without the constraints of time or geography. For example, Kashian and Jacobson (30) found that sharing in medical online support groups may improve participants’ physical and mental health, as well as their overall quality of life.

Another study demonstrated that members of online support groups encourage one another through writing optimistic comments, consulting with others, sharing their own experiences, tagging friends who can help, and using reactions such as likes and emojis to respond to posts (31). These groups offer all members an equal opportunity to express opinions and emotions, ask questions, share and receive information, and propose solutions or advice to address the challenges raised within the group (32, 33).

Studies show that social media use fulfills key social functions, including maintaining relationships, fostering belonging, and strengthening perceived social support through interaction with like-minded others (34). Extending this view, research has demonstrated that following collective trauma, social media platforms can serve as spaces for resilience-building, where humor-based interaction supports emotional coping and communal connection during war (35).

Recent research has increasingly focused on Facebook groups in general, and on Facebook support groups in particular (36, 37). Facebook communities play a significant role in providing emotional support and sharing information with their members. They create diverse spaces for discussion, promote community engagement, enable a continuous flow of information, and reinforce a sense of community (38). For instance, online parenting communities meet the need for emotional support and information among first-time parents and help alleviate and manage their emotional experiences (39).

### 2.3 Research rationale

The present study specifically focuses on Facebook-based ADHD support groups for adults with ADHD. While previous research has highlighted the role of online groups in providing informational and emotional resources, little attention has been paid to ADHD-specific communities and their unique dynamics. Moreover, the link between participation in these groups and the mental well-being of adults with ADHD has remained largely unexplored.

By examining participants’ motivations for joining ADHD-focused Facebook groups, their perceived value, and the types of support they receive, this research aims to generate a nuanced understanding of how engagement in online groups contributes to informational, emotional, and social support. In particular, the study explores how such support may alleviate psychosocial challenges commonly associated with ADHD such as feelings of failure, rejection, and stress and ultimately enhance participants’ overall well-being.

This focus addresses a critical gap in literature: whereas existing studies often examine social support in ADHD or the use of Facebook groups for other health conditions, the intersection of these two areas has not been systematically investigated yet, as far as we know. By integrating the perspectives of social support theory, and ADHD research, the present study offers an innovative contribution that situates ADHD support within the broader context of digital mental health and well-being. In addition, identifying how these groups function may inform the development of tailored digital tools and interventions to better support individuals with ADHD.

In line with this rationale, the study addresses the following research questions:

- 1. What are the motivations for joining Facebook groups dedicated to ADHD?
- 2. How do personal narratives or shared experiences within ADHD-focused Facebook groups affect participants on a personal level?
- 3. In what ways do members of ADHD-focused Facebook groups perceive these groups as sources of informational support?
- 4. In what ways do members of ADHD-focused Facebook groups perceive these groups as sources of emotional support?

## 3 Method

This study focuses on exploring the experiences, challenges, and perceived significance of Facebook attention-support groups for individuals with ADHD. The empirical material is based on twenty semi-structured interviews conducted in November 2024, providing in-depth insights into participants’ perspectives and the role these groups play in their daily lives.

### 3.1 Sampling population

The study sample comprised 20 participants drawn from Facebook attention groups. The group consisted of 11 women and 9 men, all of whom were above the age of 18. Participants’ ages ranged from 27 to 64 years (M = 42.15, SD = 10.25).

### 3.2 Data collection

A call for participation was posted in ADHD Facebook groups, with approval obtained from the group administrators for closed groups. Data were collected through semi-structured interviews designed to elicit participants’ personal experiences, with a focus on how Facebook attention groups provide emotional and informational support. Prior to participation, all individuals provided informed consent, confirming their voluntary involvement. The interviews were conducted via the Zoom videoconferencing platform, allowing synchronous interaction at a time and setting convenient for participants. With participants’ permission, all sessions were audio-recorded and transcribed verbatim to ensure accuracy in analysis. Ethical approval for the study was obtained from the Faculty’s Institutional Review Board (IRB). The interview protocol consisted of open-ended questions addressing participants’ motivations for joining the groups, as well as the benefits and types of support they received. The interview protocol can be found in **Appendix 1**.

### 3.3 Analytic procedure for qualitative analysis

Interview data were analyzed using an inductive thematic approach, allowing findings to emerge from the dataset rather than being guided by priori theoretical assumptions (40). Inductive coding involves a “bottom-up” approach, starting directly from the data, where Codes and, eventually, themes are generated from what emerges within the data itself, which forms the foundation for interpreting and understanding its meaning (41).

The analytic process consisted of repeated readings of the transcripts, the identification and coding of recurrent patterns, and the organization of these codes into initial categories. Through iterative refinement, broader themes and sub-themes were generated. Constant comparison across categories and themes was employed to refine distinctions and to identify relationships between them. The process continues until saturation is reached, at which point no new attributes, variations, or relationships within the theoretical categories emerge (41).

Selecting an approach that emphasizes reaching saturation also serves to enhance the study’s credibility and reliability (42). In the qualitative approach, validity lies in understanding, in the creation of meaning, and in interpretation that holds significance beyond the studied subjects. This provides a conceptual framework through which the researched experience can be more fully understood (43).

### 3.4 Data reliability and trustworthiness

Several measures were taken to ensure the trustworthiness of the data. Interviews were audio-recorded and transcribed verbatim, and repeated readings supported accurate coding and theme development. Data analysis followed an inductive thematic approach, incorporating constant comparison and iterative refinement of categories, and progressed from holistic readings of the interviews to the identification and structuring of themes (44, 45). Thematic saturation was reached when no new insights emerged (46). This approach is consistent with the procedures outlined by Dolev-Cohen and Ben Israel (47), who emphasized multi-stage coding, careful documentation, and reflexive interpretation to strengthen the credibility and trustworthiness of qualitative findings.

## 4 Findings and discussion

**Figure 1:**
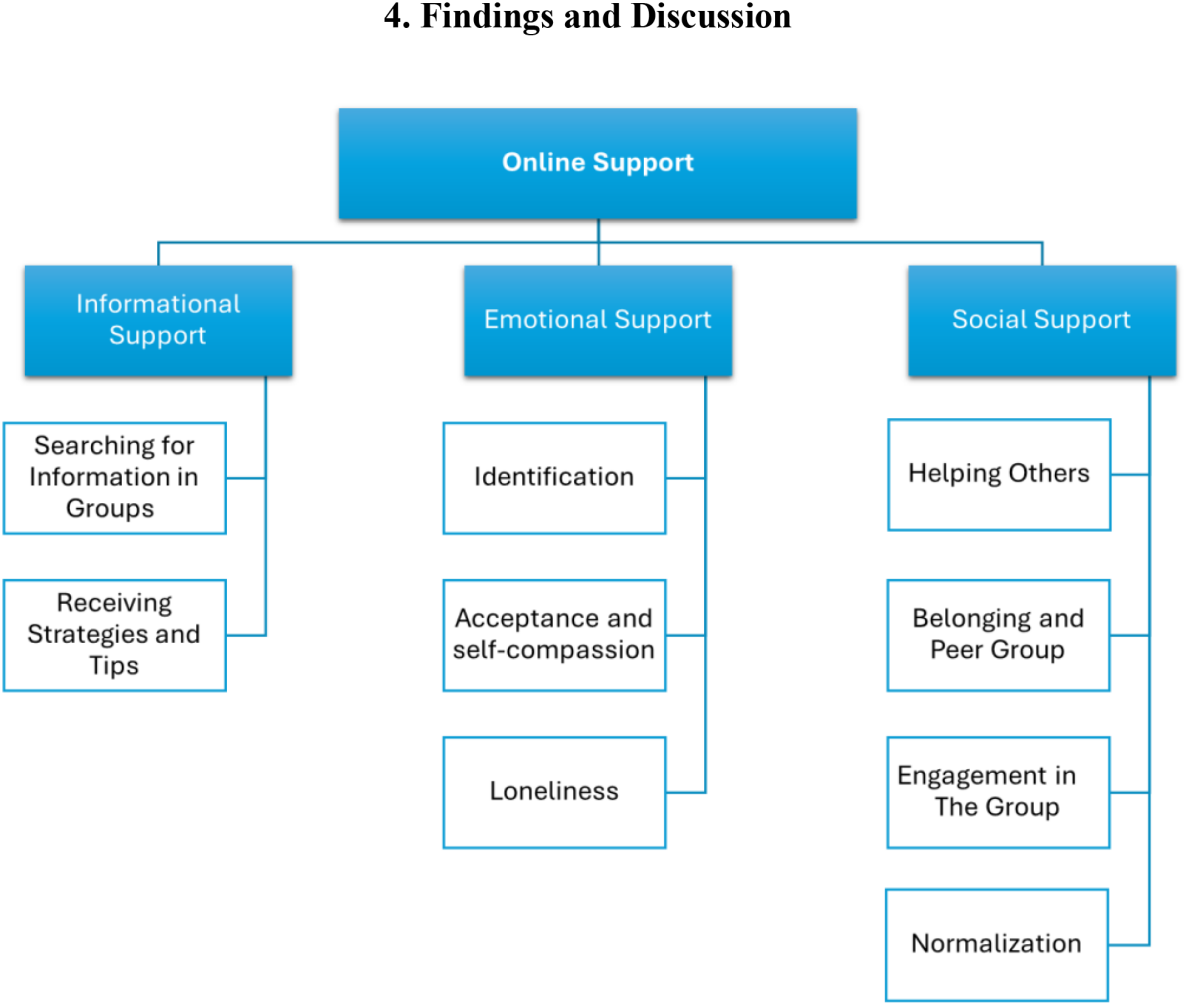
Themes and sub-themes emerging from qualitative analysis of online support in ADHD Facebook groups.

**Figure 1** presents the themes and sub-themes of Online Support in ADHD Facebook groups, demonstrating the interrelationships between the primary category and its sub-categories. **Table 1** presents a summary of these categories and sub-categories as identified in the open-ended questions, provides a brief explanation of each pattern, and includes a representative quote for each.

**Table 1:** A summary of the categories and sub-categories identified in the open-ended questions, a brief explanation of each pattern, and a representative quote:.

Category	Interpretive summaries	Representative quote
Informational support		
Searching for information in groups	The participants described turning to online groups as a central strategy for seeking practical, reliable, and accessible information.	“*There’s good knowledge there, whether it’s about a medication shortage or some treatment method or something like that. It’s amazing; everything is always there, and someone has thought about everything before.*” (H., female, 30)
Receiving strategies and tips	Participants described receiving practical strategies and tips as informational support that aids in daily coping.	“*It might be that if I had encountered a group like this earlier, it would have helped me understand myself a bit sooner, and I would have saved myself about a year in my degree or, like, I would have learned strategies that I didn’t even know existed for work, studies, and all kinds of methods.*” (E., male, 44)
Emotional support		
Identification	ADHD Facebook groups foster a strong sense of identification, as members recognize themselves in the experiences and stories of others.	“*For me, it had tremendous value. I joined one group, ‘okay, let’s see what this is,’ and I got hooked, then I joined every possible group because suddenly you read so many things that people experience or do or go through, and you say, ‘Ah yes, me too.*” (A., female, 27)
Acceptance and self-compassion	ADHD Facebook groups promote self-compassion and reduce self-criticism through a supportive group environment.	*“To say where I overcome and cope, and where I give myself a break, not out of despair, but while accepting myself.”* (D., male, 56)
Loneliness	ADHD Facebook groups provide a supportive space for sharing experiences and receiving understanding, reducing feelings of loneliness.	*“It’s hard to believe this happened to so many people, and it’s like, it’s part of this thing, and I’m not alone in it.”* (A., female, 27)
Social support		
Helping others	Helping others in ADHD Facebook groups fosters connection, purpose, and mutual support through the sharing of experiences and knowledge.	*“To me, it’s somewhat of a mission to be there for other people with ADHD.”* (D., male, 56)
Belonging and peer group	ADHD Facebook groups offer a sense of belonging and a supportive space for open dialogue that aids coping with daily challenges.	*“Seeing that we are in groups of equals is probably very comforting, being part of a tribe rather than a lone wolf, because in my own social circle, I am always different, not weird, but they always said I was special, though not necessarily in a very positive connotation. Here, it’s very pleasant to be in that kind of family of troubles.”* (G. female, 64)
Engagement in the group	Engagement in ADHD Facebook groups fosters knowledge sharing, mutual support, and a sense of value, empowering the community.	*“Right after I got diagnosed, that was really a whole year, these were my groups because I entered them all the time and I was very responsive, very active, trying to learn a lot.”* (A. female, 27)
Normalization	ADHD Facebook groups help members normalize their challenges, fostering acceptance and reducing feelings of isolation.	*“It was actually nice to see that I’m not the only crazy one this happens to.”* (Y. male, 40)

The thematic analysis of the interviews indicated that ADHD-related Facebook groups serve as hubs for informational, emotional, and social support. These three types of support emerged from the interview data (see **Figure 1**).

### 4.1 Informational support

The interviews reveal that Facebook attention-related groups provide substantial informational support, enabling their members to access knowledge, strategies and tips, ask questions, and learn from the experiences of others. Participants expand their understanding and receive guidance regarding diagnosis, treatments, and day-to-day coping. For example:

*“If not for social media, if not for the attention communities … all the knowledge … I am living in a golden age.”* (M., female, 51)

This statement underscores the importance of social media in general and Facebook attention groups in particular as a vital source of learning and personal development. M.’s words reflect the sense of empowerment that comes from exposure to specialized and professional knowledge shared by individuals who face similar challenges.

These findings resonate with prior research demonstrating that online support groups provide essential resources for individuals coping with diverse health conditions. For instance, studies on groups devoted to gestational diabetes, miscarriage, and myositis reveal that participants frequently both seek and offer informational as well as emotional support (48–50).

Similarly, in the context of ADHD, (51) found that following a diagnosis in adulthood, many participants showed interest in learning about their condition and actively sought information through pamphlets, books, and online sources. Furthermore, adults with ADHD appear to be interested in learning about many different aspects of ADHD, and a lack of knowledge on the subject may contribute to feelings of distress (52).

Reflecting these findings, this category includes two sub-categories, as identified through the analysis of the interviews and illustrated in the themes and sub-themes in **Figure 1**: Searching for information in groups and receiving strategies and tips.

#### 4.1.1 Searching for information in groups

The interviews reveal that ADHD groups on Facebook provide rich and in-depth knowledge about ADHD and allow participants to learn from others’ personal experiences. Group members seek information on treatments and medications, deepen their understanding of their unique difficulties, and discover new coping methods through open dialogue and knowledge exchange. Within the online space, the interviewees describe ADHD groups as accessible sources of information that respond to questions as they arise, right in the moment of need. For example:

*“It’s good as a knowledge source for specific questions that suddenly come up or if you want to get some information. Helpful, helpful … There’s good knowledge there, whether it’s about a medication shortage or some treatment method or something like that. It’s amazing; everything is always there, and someone has thought about everything before.”* (H., female, 30)

H.’s remarks emphasize the sense of security that stems from knowing that no matter what question arises, someone has already dealt with it. The knowledge is experienced as a human response that advances one step forward. It seems that Facebook groups constitute a significant source for obtaining up-to-date, focused, and accessible information for people coping with attention difficulties. Group members use the platform to search for answers to concrete questions, recommendations for treatment methods, consultations regarding medications, and more. H. (30) describes the group as a rich and available source of knowledge that offers answers to nearly every question. Her description illustrates not only the practical utility of the group but also the emotional reassurance it provides. Her words highlight the experience of finding accurate and rapid answers to immediate needs and the feeling that the online community is a space where knowledge has already been collected, tested, and interpreted for those who need it, sometimes even before the question itself has fully formed. These findings correspond with research studies showing that various Facebook groups enable the creation and establishment of virtual communities with shared interests, producing discussions and activities that reflect the interests of the group members (32, 37, 53). People choose to participate in these groups to obtain information on various topics (54, 55).

Findings from the interviews suggest that online ADHD groups provide anticipatory support, giving participants quick access to collective knowledge and practical guidance. For the interviewees, this immediate, experience-based resource offers a depth and breadth of information that is difficult to achieve in offline settings.

#### 4.1.2 Receiving strategies and tips

Some of the interviewees reported that they receive practical tips and methods to improve executive functions such as time management, task planning, and organization, enabling them to cope more effectively with daily challenges. Through these tools, they deepen their understanding of how ADHD operates, set realistic expectations for themselves, and develop personalized coping strategies. For example:

*“It might be that if I had encountered a group like this earlier, it would have helped me understand myself a bit sooner, and I would have saved myself about a year in my degree or, like, I would have learned strategies that I didn’t even know existed for work, studies, and all kinds of methods.”* (E., male, 44)

It appears that one of the main expressions of informational support in Facebook groups involves the accessibility of strategies, tools, and tips that assist group members in their daily coping with attention challenges, both in studies and at work. E. (44) shared that early exposure to such a group could have brought significant changes to his academic path and personal development. His words illustrate how the knowledge accumulated and shared within the group framework is not only technical or practical but can also stimulate processes of self-insight, establish a sense of capability, and substantially influence choices and processes in academic and professional life. These findings align with research literature showing that various Facebook groups provide opportunities to share tips, resources, and recommendations on topics such as parenting, child development, health, and more, relying on real-life experiences (56, 57).

These findings highlight the unique value of online ADHD communities, where participants can access timely, peer-generated strategies and support. Virtual space allows for immediate guidance, reflection, and normalization, benefits that are often harder to achieve in offline settings.

### 4.2 Emotional support

The interviews suggest that beyond informational aspects, ADHD Facebook groups may also provide their members with meaningful emotional support. The interviews reveal that for many, the group serves as a safe space for emotional expression and sharing difficult experiences, where the act of writing and receiving supportive responses offers comfort and acceptance. Group members can vent their feelings and receive encouragement and understanding from others facing similar challenges. The sense of belonging and identification helps reduce loneliness and creates a network of support and encouragement for coping with daily challenges. For example:

*“There are days when I’m completely broken, and I write some post, and people tell you it’s not so bad or that it will pass or that they also have days like that.”* (Yu., male, 43)

*“These groups are naturally very supportive because everyone there is dealing with the same things. I see that people are mainly looking for a sympathetic ear or for someone to reassure them that they’re okay.”* (M., female, 43)

The statements of Y. and M. emphasize the emotional support in ADHD groups, which allows their members to feel less isolated and to experience care and emotional solidarity, something particularly important for those coping with ADHD. These personal accounts align with and extend existing research, as the findings from the interviews support previous results known in the research literature, which in recent years has focused on Facebook groups in general and support groups on Facebook in particular (36). This is reflected in the role of participants in giving and receiving social support from their peers both informational and emotional (58–60).

Finally, based on the interview findings, this category includes three subcategories, derived from the processing and analysis of the interviews, and reflected in the themes and sub-themes shown in **Figure 1**: Identification, acceptance and self-compassion, and loneliness.

#### 4.2.1 Identification

The interviews with participants indicate that ADHD Facebook groups create a deep sense of identification, where members recognize themselves in the stories and experiences of others. Authentic sharing enables understanding that “I am not alone” and provides emotional relief in knowing that others face similar challenges. For example:

*“For me, it had tremendous value. I joined one group, ‘okay, let’s see what this is,’ and I got hooked, then I joined every possible group because suddenly you read so many things that people experience or do or go through, and you say, ‘Ah yes, me too’.”* (A., female, 27)

“*If it’s a day when you feel like a failure and haven’t accomplished anything because you’ve been procrastinating all your tasks for about two months and everything is about to collapse, then it’s like taking a breath and saying, ‘Okay, at least I’m not that bad.’ Like, there are a few other people who are really bad with me, so maybe together we’re not the worst in the world.*” (H., female, 30)

The interviews reveal that participants feel substantial relief and validation through recognizing themselves in others’ experiences. This sense of shared understanding appears to contribute to improved well-being, as members perceive their struggles as normalized and validated. Academic literature supports this link: identification predicts mental well-being by reducing feelings of loneliness (61).

In a study conducted on Facebook book clubs, a qualitative research method was used. The study’s findings indicated a sense of identification that allowed group members to discover aspects within themselves and understand that they are not alone (62). This is particularly relevant because, like our findings, identification emerged as a key component of the participants’ deep therapeutic process, highlighting its central role in fostering emotional insight and well-being. Based on the research findings, ADHD Facebook groups foster a sense of identification among members, contributing to emotional processing and the development of a collective identity. This highlights the importance of these groups as psycho-social spaces that support participants’ well-being.

#### 4.2.2 Acceptance and self-compassion

The interviews reveal that ADHD Facebook groups enable individuals to view themselves more forgivingly and acceptingly, fostering self-compassion while reducing self-criticism and internal judgment. Participants indicated that these attitudes were influenced by the supportive group environment. For example:

*“To say where I overcome and cope, and where I give myself a break, not out of despair, but while accepting myself.”* (D., male, 56)

*“Today I understand that it’s something that happens, and I don’t beat myself up about it. Sometimes I manage to cope better, sometimes less, but I understand what is going on and accept that it’s okay.”* (Ye., male, 32)

*“It allows me to be a bit more empathetic and a bit more forgiving toward these things in myself.”* (T., female, 47)

Self-compassion emerged as a recurring theme in the participants’ statements, describing an emotional process of self-acceptance and non-self-punishment considering attention difficulties. ADHD Facebook groups contribute to this process by providing an empathic and supportive space, where participants are exposed to stories like their own and learn about different ways to cope with challenges.

Similarly, sharing personal stories within online support groups creates an empathetic environment that promotes emotional healing. Hearing about the experiences of others who have navigated comparable challenges can offer emotional comfort and validation, helping patients feel understood and less isolated. For example, knowing that others have successfully managed similar difficulties can reduce fear and anxiety regarding one’s own condition (58, 59).

The sense of belonging and non-judgmental atmosphere characteristic of these groups enables members to see themselves with softer eyes as people learning to live with difficulties in an accepting and inclusive manner. Academic literature also acknowledges the importance of supporting online communities in fostering self-compassion. For instance, in a study by Seekis et al. (63), a safe online Facebook community where women shared their experiences in the context of a brief mindful self-compassion program improved body image and self-compassion and served as an effective strategy to foster a positive relationship with the body and self.

It appears that through emotional support, normalization of experiences, development of mutual empathy, and strengthening of self-efficacy, Facebook support groups provide an environment that cultivates less self-compassion deficit, that is, a reduction in participants’ self-criticism and judgmental attitudes.

#### 4.2.3 Loneliness

Several interviewees reported that ADHD Facebook groups create a space where people can share their experiences, receive understanding responses, and feel that they are not alone in their struggles. The opportunity to read similar stories, converse with others, and gain non-judgmental listening reduces feelings of loneliness. It appears that participants described emotional relief stemming from the understanding that they are part of a large group facing similar challenges.

One of the participants shares her sense of relief upon discovering the commonality between her experiences and those of other group members:

*“It’s hard to believe this happened to so many people, and it’s like, it’s part of this thing, and I’m not alone in it.”* (A., female, 27)

Another participant also notes the great benefit of knowing she is not alone with her personal experiences:

*“Sometimes it’s just good and nice to know and hear that you’re not alone with your experiences.”* (Sh., female, 35)

These statements reflect the importance of the space created within ADHD Facebook groups, which reduces feelings of loneliness and enables group members to feel genuine and empowering emotional support. This aligns with previous literature, For example, Prescott et al. (64) found that Facebook groups provide online support that can enhance a sense of connection, reduce loneliness, offer a platform for perspective comparison related to personal experiences, foster hope, and increase empowerment among users who utilize Facebook groups as a support mechanism, thereby contributing to mental well-being. However, the broader impact of social media on mental health and well-being remains debated. Some research suggests that social media can help people sustain their social connections, serving as a strategy to cope with loneliness and psychological distress (65, 66). Conversely, other studies indicate that higher engagement with social media in general may be associated with poorer mental health outcomes (67, 68) and increased feelings of loneliness (69). Therefore, the extent to which social media use can effectively alleviate stress remains uncertain.

Taking together, these findings highlight that ADHD Facebook groups may offer a unique and meaningful space for social connection, distinct from general social media use. From the shared narratives, the mere possibility of speaking openly and discovering that others face similar experiences reduces loneliness and offers comfort and emotional support.

### 4.3 Social support

The interviews indicate that ADHD Facebook groups foster a deep sense of belonging by connecting members with peers who face similar challenges. Participation in the group allows individuals to share, respond, and feel part of a community that understands them without needing to explain their ADHD-related difficulties. Through open dialogue and shared experience, many members experience normalization of their feelings, which reduces the sense of being an outsider and provides them with confidence in their daily coping.

These findings echo a study by Ya'ari et al. (70), in which participants emphasized that the minority status of their parental identity often resulted in feelings of isolation and social alienation, motivating them to seek out online support groups. In line with this, participants who are parents of only one child reported seeking out a Facebook support group on this topic, as the marginality of their parental identity often led to feelings of isolation and alienation. The group’s structure enabled them to meet other parents with a similar reproductive status, alleviating feelings of social abnormality and fostering a sense of normalization (70).

This category comprises four subcategories, as derived from the processing and analysis of the interviews and reflected in the themes and sub-themes in **Figure 1**: Helping others, belonging and peer group, engagement in the group, and normalization.

#### 4.3.1 Helping others

The interviews reveal that assisting others in ADHD Facebook groups fosters connection through the sharing of experiences and knowledge, enabling them to find support and solutions. This reciprocal exchange means that each piece of advice or encouragement not only benefits the recipient but also reinforces the giver’s sense of purpose and meaning. For example, Gal et al. (71), found that Facebook community administrators experience a sense of fulfillment from helping others. They reported that managing the community contributes to their personal development and serves as a source of pride and self-expression. When the community acts collectively, it becomes an empowering space that provides members with strength and tools to cope with various challenges. Supporting this, the study by Prescott et al. (64) found that group members are not only recipients of help but also actively function as a “human resource” for others, often feeling motivated to share the knowledge and experiences they have accumulated to assist fellow members.

One participant shared her experience of frequently providing support to other group members because of the expertise she has gained in the field:

*“I often find myself responding because I’ve researched this area and it’s very much on my mind, so sometimes I have answers and good advice, and I really feel the need to stop everything and respond.”* (L., female, 28)

Another participant described a sense of mission that motivated him to help others:

*“To me, it’s somewhat of a mission to be there for other people with ADHD.”* (D., male, 56)

These statements highlight the dynamic of mutual support within Facebook groups, where members not only feel the need for assistance but are also willing to invest effort and actively help others.

Literature shows that group members assist each other in various ways. For example, members of support groups encourage one another by writing optimistic comments, consulting with each other, sharing personal experiences, tagging members who can provide help, and reacting with likes and emotional responses to posts (31). Additionally, a Canadian study examining the use of online support groups for caregivers of children and youth with complex care needs highlighted the importance of altruistic contribution and showed that participants expressed a desire to help others going through similar situations by sharing the knowledge they had gained, and that emotional posts generated the highest level of engagement (72). In another study, it was found that participants became accustomed to regularly helping other participants, as one participant noted: “I’m really adept at helping them with their problems, as we don’t see each other often, and social media is a really helpful platform” (73, p.425).

The findings of the present study underscore the active and empowering potential of the participants themselves, who experience a sense of value, mission, and community connection through giving. A key contribution of this study is its demonstration that giving and helping others does not necessarily stem from personal stability but can arise from the challenges individuals have faced.

For participants coping with attention challenges, the act of supporting others is not a secondary outcome but a central means of coping for themselves. In this sense, giving functions as a reciprocal process: while it benefits the community, it simultaneously strengthens the giver’s own sense of capability, meaning, and belonging. This dual role of giving, born out of struggle yet reinforcing resilience, adds an innovative perspective to existing understandings of online support.

#### 4.3.2 Belonging and peer group

The interviews reveal that ADHD Facebook groups provide a space where individuals experience a sense of belonging within a peer group, surrounded by others who share similar experiences. Knowing that a supportive community exists instills confidence and enables open, sincere dialogue that helps members cope with daily challenges.

For example, a participant shared her feeling of comfort in groups where she senses belonging:

*“Seeing that we are in groups of equals is probably very comforting, being part of a tribe rather than a lone wolf, because in my own social circle, I am always different, not weird, but they always said I was special, though not necessarily in a very positive connotation. Here, it’s very pleasant to be in that kind of family of troubles.”* (G, female, 64)

Similarly, another participant describes the need for belonging, particularly highlighting the difference between other groups and the ADHD Facebook group:

*“People are searching … searching for belonging to this peer group … even if it’s virtual. They can provide us with something … that we don’t have with close friends. We don’t have it in the family. We don’t have it at work. Not everyone can understand us or our difficulties, but here, you are understood.”* (H, female, 34)

Statements like those of G and H emphasize the advantage of a supportive group based on shared experiences, where members can feel genuine belonging, thereby strengthening mutual understanding and acceptance. These findings are consistent with prior research demonstrating that online groups may serve as gateways to finding similar people who provide social support and identity affirmation. For instance, in Godard et al.’s (74) research, participants described these online groups as supportive communities and generally viewed them positively, highlighting opportunities for laughter, learning, and a sense of belonging. Another study by Gazit et al. (62) found that active participation in online reading groups on Facebook allowed members sharing deep emotions evoked during reading and feeling a sense of belonging through these shared experiences.

Taken together, the current findings provide new insight into the central role of the sense of belonging created within ADHD Facebook groups, which acts as a significant and reinforcing factor for participants. Unlike other environments where members may experience alienation or misunderstanding, the group functions as a warm and supportive community and serves as a true peer group. The sense of belonging that emerges is not only derived from identification with shared experiences but also from the way the group enables authentic emotional expression without fear of judgment. Thus, it contributes not only to social connectedness but also to the development of self-worth and the enhancement of mental well-being.

#### 4.3.3 Engagement in the group

The interviews indicate that engagement in ADHD Facebook groups creates a dynamic space where members share experiences, exchange knowledge, and offer new perspectives. By responding to posts, asking questions, suggesting solutions, and supporting one another, members contribute to a vital source of learning and insights. Open dialogue and collective activity not only benefit all participants but also enhance everyone’s sense of value and meaning, contributing to the empowerment of the entire community. For example, a study showed that Facebook groups provide a unique platform for patients to share experiences, expand their support networks, and build a community, which in turn enhances their quality of life (75).

Interviewees report that they do not merely read posts but actively respond and participate in discussions, enriching the group’s dynamics.

A participant describes her strong engagement with the group after receiving her diagnosis, devoting considerable time to getting acquainted and frequently responding:

*“Right after I got diagnosed, that was really a whole year, these were my groups because I entered them all the time and I was very responsive, very active, trying to learn a lot.”* (A, female, 27)

A participant notes his intensive engagement in the group, where he not only reads but also actively comments:

*“I would look, read, comment because I had something to say, and it turns out I’m very active there.”* (D, male, 56)

Their statements illustrate the importance of consistent engagement within the group, which contributes to the experience of social support. Previous studies emphasize that Facebook communities play a significant role in providing support and information to their members. These communities create various discussion spaces, foster community engagement, enable continuous information flow, and strengthen the sense of belonging (76). For example, online parenting communities address the need for support and information experienced by first-time parents and help alleviate and manage those emotions (39).

The current study’s findings suggest that active engagement in ADHD Facebook groups is a key component of the community belonging experience. Rather than passively consuming content, participants engage in mutual sharing of knowledge, experiences, and emotional support. Through Open dialogue, encouraging responses, and proposed solutions create a sense of personal value and deepen the mutual responsibility among group members. This active participation not only meets personal needs but also contributes to identity formation, strengthens self-efficacy, and enables members to view themselves as contributors and influencers rather than passive recipients.

These findings correspond with previous research showing that active participation in online groups is relatively rare. Most users read content without contributing and are therefore referred to as lurkers. However, studies suggest that the extent of engagement is not random but shaped by various personal and environmental factors. For example, Gazit et al. (77) found that both personality and contextual factors jointly influence whether individuals become active participants or lurkers in online communities. Similarly, Amichai-Hamburger et al. (78) identified personal, social, and technological factors affecting online participation levels. These findings support the idea that meaningful, active involvement in ADHD Facebook groups reflects broader psychological and social mechanisms that encourage connection, contribution, and mutual support in digital communities.

#### 4.3.4 Normalization

Various interviewees reported that ADHD Facebook groups help them perceive the difficulties or challenges they face as normal and common situations, creating a sense of acceptance and understanding that reduces feelings of isolation and aids in achieving a new, calm perspective on coping with challenges.

The interviews reveal that ADHD Facebook groups provide their members with a sense of normalization by allowing them to view personal difficulties as part of the broader experience shared by many others. This normalization helps reduce feelings of loneliness and difference, reinforcing the understanding that ADHD experiences are neither unique nor exceptional.

A participant shares the relief he felt upon realizing he was not alone in his struggles:

*“It was actually nice to see that I’m not the only crazy one this happens to.”* (Yo, male, 40)

Another participant talks about how the recognition he received in the groups changed his self-perception, as he came to understand that nothing is inherently wrong with him:

“Something was wrong with me, and suddenly, wow, there’s nothing wrong with you, bro, look how many are like you. You’re not alone.” (Ya, male, 35)

A participant explains the relief she feels when she understands she is not an outlier but part of a normal group facing similar challenges:

*“It’s always nice to see that I’m not an outlier but really, really normal in a group with this disorder.”* (G, female, 64)

These statements emphasize the contribution of ADHD Facebook groups in reducing feelings of difference and creating a sense of normalization and shared experience among others facing similar challenges.

There appears to be a convergence between the interview findings and previous literature. For example, studies have shown that users engage with social media in various ways such as creating social connections, searching and sharing information, entertainment, and following content from other users. These uses help participants feel a sense of belonging, maintain interpersonal relationships, and encounters with like-minded users strengthen the sense of social support (34). Consistent with this, the way the group was organized enabled participants to connect with other parents in comparable reproductive situations, alleviating feelings of social isolation and fostering a sense of normalcy (70). Similarly, Naveh and Bronstein (79) found that in a virtual community for pregnant women with diabetes, group members were able to find meaning in their changing realities and co-construct a sense of normality through conversations.

This finding highlights the unique contribution of ADHD Facebook groups not only as spaces for social support but also as mechanisms for normalizing experiences among individuals coping with ADHD. The sense of normalization arising from shared discourse and identification with similar experiences plays a significant role in reducing feelings of difference and isolation and encourages a new, more reconciled interpretation of personal challenges.

## 5 Conclusions

ADHD-focused Facebook groups emerged as spaces of connection and support, where members share experiences, exchange practical knowledge, and feel included. These groups allow for authentic, open, and nonjudgmental expression, playing a significant role as sources of informational, emotional, and social support. Members share practical knowledge, such as time management strategies, digital tools, professional recommendations, and lived insights, in an egalitarian and accessible manner. Beyond sharing information, the groups serve as emotionally supportive environments where participants can feel seen and that they belong. Reading about others’ similar experiences, responding, relating, and receiving support helps generate a sense of normalization, which many participants described as essential in reducing feelings of alienation and loneliness. The interviews suggest that ADHD-focused Facebook groups function as islands of belonging and identification for individuals with ADHD. Importantly, these findings also point to a deeper layer of online support, beyond the well-documented technical affordances of digital platforms such as accessibility, anonymity, and immediacy. As demonstrated in studies of online support for people who stutter (80), mediated environments can enable help-seeking itself in situations where offline interaction is perceived as difficult or even impossible. Similarly, for individuals with ADHD whose condition is often associated with social isolation and barriers to offline support-seeking, the online space does not merely function as a technical substitute for face-to-face interaction but enables access to support that may otherwise be unavailable. Their role extends beyond that of mere “online discourse arenas,” transforming them into vital resources and agents of change that enhance the quality of life for those with ADHD. In this sense, the contribution of these groups lies not only in reducing loneliness as a general emotional state but in bridging a structural social gap rooted in the disorder itself. As such, these groups illustrate the human and social potential embedded in social media in general, and in ADHD-focused Facebook communities in particular, offering an inclusive, compassionate, and empowering space.

## 6 Limitations and future research

While this study contributes to understanding ADHD-focused Facebook groups as spaces of connection and support, several limitations should be noted. First, the qualitative interview-based approach involves subjective interpretation, which, while capturing participants’ voices in depth, limits generalizability. Second, the findings may not fully capture the diversity of experiences among ADHD group members and in future studies, it may be useful to distinguish between different age ranges, such as younger and older adults, or different generational cohorts. Third, the examined groups constitute unique communities, and findings may not apply to other online groups or cultural contexts. Furthermore, the study focused exclusively on Israeli ADHD Facebook groups, where local socio-cultural factors may influence interactions and perceived support, and caution is therefore required when extending the findings to other countries or platforms.

This study demonstrates the critical role of ADHD-focused Facebook groups in providing emotional, informational, and social support.

Theoretically, the findings deepen the understanding of how digital spaces enable multi-layered forms of support: emotional, social, and informational while also illuminating the mechanisms that foster belonging and validation within ADHD communities online.

Practically, the results highlight how insights from these communities can inform the development of targeted online interventions and psychoeducational programs for adults with ADHD. They also provide guidance for group administrators seeking to cultivate supportive environments and offer recommendations for mental health professionals on integrating online peer groups into broader treatment and support frameworks.

Building on these findings, future research could explore how the peer-support dynamics identified in ADHD-focused Facebook groups may be applied to the design of structured digital interventions and psychoeducational programs. Such studies could evaluate their effectiveness in enhancing well-being and fostering a sense of belonging among adults with ADHD across different cultural and technological contexts. In this context, it may also be worthwhile considering developments in synthetic support technologies. Preliminary studies have already examined the potential of bot-mediated peer support for adults with ADHD (81), and more recent work suggests that AI-generated responses may be perceived as supportive and acceptable in certain peer-support contexts, while human support remains preferred for highly sensitive issues (82), indicating promising directions for hybrid or complementary AI-based support systems.

By centering the lived experiences of adults with ADHD in online communities, this study advances theoretical and empirical understandings of digital peer support and highlights the unique role of social media in shaping well-being and belonging among neurodivergent populations.

## Data Availability

The raw data supporting the conclusions of this article will be made available by the authors, without undue reservation.

## Appendix 1—Interview protocol

The following script was used for interviews:

1. Tell me if and how you were diagnosed with ADHD, and at what age this occurred.

2. Do you currently receive any pharmacological treatment for ADHD?

3. Describe in what areas or situations in your daily life ADHD manifests itself for you.

4. Share a specific experience or example that illustrates how ADHD has affected you or your life.

5. What motivated you to join the ADHD support group?

6. In what ways does the information you are exposed to in the group contribute to you from an informational perspective?

7. In what ways does the information you are exposed to in the group affect you emotionally?

8. What do you consider to be the main advantages of the group for its members?

9. Tell me about a meaningful story or moment you experienced in the group that touched you personally.

10. What makes you choose to remain a member of the ADHD group today?

11. How do you think participating in the group has influenced your sense of self-efficacy in coping with ADHD?

## References

[1] Lambez B Harwood-Gross A Golumbic EZ Rassovsky Y . Non-pharmacological interventions for cognitive difficulties in ADHD: a systematic review and meta-analysis. J Psychiatr Res. (2020) 120:40–55. doi: 10.1016/j.jpsychires.2019.10.007, PMID: 31629998

[2] American Psychiatric Association . Diagnostic and statistical manual of mental disorders: DSM-5. 5th ed. Washington, DC: American Psychiatric Association (2013).

[3] Bunford N Evans SW Langberg JM . Emotion dysregulation is associated with social impairment among young adolescents with ADHD. J Attention Disord. (2018) 22:66–82. doi: 10.1177/1087054714527793, PMID: 24681899

[4] Bunford N Kujawa A Dyson M Olino T Klein DN . Examination of developmental pathways from preschool temperament to early adolescent ADHD symptoms through initial responsiveness to reward. Dev Psychopathol. (2021) 16:1–13. doi: 10.1177/1087054714527793, PMID: 33722319

[5] Ophir Y . ADHD is not an illness and ritalin is not a cure: A comprehensive rebuttal of the (Alleged) scientific consensus. World Scientific (2022).

[6] Drechsler R Brem S Brandeis D Grünblatt E Berger G Walitza S . ADHD: Current concepts and treatments in children and adolescents. Neuropediatrics. (2020) 51:315–35. doi: 10.1055/s-0040-1701658, PMID: 32559806 PMC7508636

[7] Ayano G Betts K Dachew BA Alati R . Academic performance in adolescent offspring of mothers with prenatal and perinatal psychiatric hospitalizations: a register-based, data linkage, cohort study. Psychiatry Res. (2023) 319:114946. doi: 10.1016/j.psychres.2022.114946, PMID: 36463723

[8] Pang X Wang H Dill SE Boswell M Pang X Singh M . Attention Deficit Hyperactivity Disorder (ADHD) among elementary students in rural China: prevalence, correlates, and consequences. J Affect Disord. (2021) 293:484–91. doi: 10.1016/j.jad.2021.06.014, PMID: 34280772

[9] Heiss R Gell S Rothlingshofer E Zoller C . How threat perceptions relate to learning and conspiracy beliefs about COVID-19: evidence from a panel study. Pers Individ Dif. (2021) 175. doi: 10.1016/j.paid.2021.110672, PMID: 33518866 PMC7825816

[10] Stevic A Schmuck D Matthes J Karsay K . Age matters’: a panel study investigating the influence of communicative and passive smartphone use on wellbeing. Behav Inf Technol. (2021) 40:176–90. doi: 10.1080/0144929X.2019.1680732

[11] Lane DS Lee SS Liang F Kim DH Shen L Weeks BE . Social media expression and the political self. J Communication. (2019) 69:49–72. doi: 10.1093/joc/jqy064

[12] Ginapp CM Greenberg NR Macdonald-Gagnon G Angarita GA Bold KW Potenza MN . The experiences of adults with ADHD in interpersonal relationships and online communities: a qualitative study. SSM - Qual Res Health. (2023) 3:100223. doi: 10.1016/j.ssmqr.2023.100223, PMID: 37539360 PMC10399076

[13] Harpin V Mazzone L Raynaud JP Kahle J Hodgkins P . Long-term outcomes of ADHD: a systematic review of self-esteem and social function. J Attention Disord. (2016) 20:295–305. doi: 10.1177/1087054713486516, PMID: 23698916

[14] Laslo-Roth R Bareket-Bojmel L Margalit M . Loneliness experience during distance learning among college students with ADHD: the mediating role of perceived support and hope. Eur J Special Needs Educ. (2022) 37:220–34. doi: 10.1080/08856257.2020.1862339

[15] Riglin L Leppert B Dardani C Thapar AK Rice F O'Donovan MC . ADHD and depression: investigating a causal explanation. psychol Med. (2021) 51:1890–7. doi: 10.1017/S0033291720000665, PMID: 32249726 PMC8381237

[16] Chewning LV Montemurro B . The structure of support: Mapping network evolution in an online support group. Comput Hum Behav. (2016) 64:355–65. doi: 10.1016/j.chb.2016.07.006

[17] Nystrom A Petersson K Janlöv AC . Being different but striving to seem normal: the lived experiences of people aged 50+ with ADHD. Issues Ment Health Nurs. (2020) 41:476–85. doi: 10.1080/01612840.2019.1695029, PMID: 32267788

[18] Ek A Isaksson G . How adults with ADHD get engaged in and perform everyday activities. Scandinavian J Occup Ther. (2013) 20:282–91. doi: 10.3109/11038128.2013.799226, PMID: 23713791

[19] Schreuer N Dorot R . Experiences of employed women with attention deficit hyperactive disorder: a phenomenological study. Work. (2017) 56:429–41. doi: 10.3233/WOR-172509, PMID: 28269805

[20] Guntuku SC Ramsay JR Merchant RM Ungar LH . Language of ADHD in adults on social media. J Attention Disord. (2019) 23:1475–85. doi: 10.1177/1087054717738083, PMID: 29115168

[21] Atique J Weerawardhena H Climie EA Callahan BL . Distracted, hyperactive, and thriving factors supporting everyday functioning in adults with ADHD. BMC Psychiatry. (2025) 25:1–12:418. doi: 10.1186/s12888-025-06804-5, PMID: 40269791 PMC12020315

[22] Reeble CJ Lefler EK Abu-Ramadan T Bodalski EA Canu WH . Social support in college students with ADHD symptoms: quantity beats quality in moderating impairment. J Coll Student Ment Health. (2024) 38:519–40. doi: 10.1080/87568225.2023.2202351

[23] Powell V Riglin L Hammerton G Eyre O Martin J Anney R . What explains the link between childhood ADHD and adolescent depression? Investigating the role of peer relationships and academic attainment. Eur Child Adolesc Psychiatry. (2020) 29:1581–91. doi: 10.1007/s00787-019-01463-w, PMID: 31932968 PMC7595988

[24] Ogg JA Bateman L Dedrick RF Suldo SM . The relationship between life satisfaction and ADHD symptoms in middle school students: using a bifactor model. J Attention Disord. (2016) 20:390–9. doi: 10.1177/1087054714521292, PMID: 24514584

[25] Krauss A Schellenberg C . ADHD symptoms and health-related quality of life of adolescents and young adults. Eur J Health Psychol. (2022) 29:165–74. doi: 10.1027/2512-8442/a000104

[26] Simmons JA Antshel KM . Bullying and depression in youth with ADHD: a systematic review. Child Youth Care Forum. (2021) 50:379–414. doi: 10.1007/s10566-020-09586-x

[27] Beaton DM Sirois F Milne E . The role of self-compassion in the mental health of adults with ADHD. J Clin Psychol. (2022) 78:2497–512. doi: 10.1002/jclp.23354, PMID: 35334113 PMC9790285

[28] Amichai-Hamburger Y Hayat T . Social networking. In: Rössler P , editor. The international encyclopedia of media effects. Wiley, Hoboken, NJ (2017). p. 1–12.

[29] Statista . Global social networks ranked by number of users 2024 (2024). Available online at: https://www.statista.com/statistics/272014/global-social-networks-ranked-by-number-of-users/ (Accessed November 5, 2025).

[30] Kashian N Jacobson S . Factors of engagement and patient-reported outcomes in a stage IV breast cancer Facebook group. Health Communication. (2018) 35:75–82. doi: 10.1080/10410236.2018.1536962, PMID: 30351185

[31] Lee G Suzuki A . Motivation for information exchange in a virtual community of practice: evidence from a Facebook group for shrimp farmers. World Dev. (2020) 125:104698. doi: 10.1016/j.worlddev.2019.104698

[32] Bar-Ilan J Gazit T Amichai-Hamburger Y . Leading factors that enhance engagement in closed Facebook groups. Inf Res. (2020) 25:866. doi: 10.47989/irpaper866

[33] Kim H Lee J Oh SE . Individual characteristics influencing the sharing of knowledge on social networking services: online identity, self-efficacy, and knowledge sharing intentions. Behav Inf Technol. (2020) 39:379–90. doi: 10.1080/0144929X.2019.1598494

[34] Alhabash S Ma M . A tale of four platforms: motivations and uses of Facebook, Twitter, Instagram, and Snapchat among college students? Soc Media + Soc. (2017) 3:1–13. doi: 10.1177/2056305117691544

[35] Steinfeld N Rosenberg H Mahat-Shamir M . TikTok war humor: social and psychological functions of humor videos by micro-influencers and ordinary users during conflict. Front Psychol. (2025) 16:1637194. doi: 10.3389/fpsyg.2025.1637194, PMID: 40927345 PMC12414762

[36] Gazit T Amichai-Hamburger Y . Factors underlying engagement in Facebook support groups of female infertility patients. psychol Rep. (2021) 124:1150–73. doi: 10.1177/0033294120934703, PMID: 32597374

[37] Avizohar C Gazit T Aharony N . Facebook medical support groups: the communication privacy management perspective. Aslib J Inf Manage. (2023) 75:664–84. doi: 10.1108/AJIM-10-2021-0298

[38] Gazit T . Exploring leadership in Facebook communities: personality traits and activities. In: Proceedings of the 54th hawaii international conference on system sciences. Virtual Conference (2021). p. 3027–36.

[39] Sjöberg M Lindgren S . Challenging the roles of ‘skilled’ professionals and ‘risky’ young mothers: peer support, expertise, and relational patterns in Facebook groups. J Technol Hum Serv. (2017) 35:247–70. doi: 10.1080/15228835.2017.1367350

[40] Glaser BG Strauss AL . Discovery of grounded theory: strategies for qualitative research. London: Taylor and Francis (1999).

[41] Willig C Rogers WS eds. The SAGE handbook of qualitative research in psychology. London: Sage (2017).

[42] Roy K Zvonkovic A Goldberg A Sharp E LaRossa R . Sampling richness and qualitative integrity: challenges for research with families. J Marriage Family. (2015) 77:243–60. doi: 10.1111/jomf.12147

[43] Kvale S . The 1,000-page question. Qual Inq. (1996) 2:275–84. doi: 10.1177/107780049600200302

[44] Giorgi A . The descriptive phenomenological psychological method. J Phenomenological Psychol. (2012) 43:3–12. doi: 10.1163/156916212X632934

[45] Spinelli E . The interpreted world: An introduction to phenomenological psychology. (2005).

[46] Guest G Bunce A Johnson L . How many interviews are enough? An experiment with data saturation and variability. Field Methods. (2006) 18:59–82. doi: 10.1177/1525822X05279903

[47] Dolev-Cohen M Ben Israel H . Teachers coping with online sexual bullying by students: A qualitative study. Psychol Violence. (2025) 15:525. doi: 10.1037/vio0000579

[48] Afsar AP Ghosh S Titus RS Cheng K Kanawala AA Kerkhof P . Content analysis of patient support groups related to myositis on Facebook. Clin Rheumatol. (2024) 43:725–32. doi: 10.1007/s10067-023-06854-8, PMID: 38212556 PMC10834555

[49] Callen S Oxlad M . Support sought and offered online for miscarriage: content analysis of a Facebook miscarriage support group. Psychol Health. (2024) 40, 2094–2113. doi: 10.1080/08870446.2024.2382790, PMID: 39039665

[50] Pham S Churruca K Ellis LA Braithwaite J . Help-seeking, support, and engagement in gestational diabetes mellitus online communities on Facebook: content analysis. JMIR Formative Res. (2024) 8. doi: 10.2196/49494, PMID: 38407949 PMC10928526

[51] Aoki Y Tsuboi T Furuno T Watanabe K Kayama M . The experiences of receiving a diagnosis of attention deficit hyperactivity disorder during adulthood in Japan: a qualitative study. BMC Psychiatry. (2020) 20:1–8. doi: 10.1186/s12888-020-02774-y, PMID: 32677922 PMC7366299

[52] Seery C Wrigley M O'Riordan F Kilbride K Bramham J . What adults with ADHD want to know: a Delphi consensus study on the psychoeducational needs of experts by experience. Health Expectations. (2022) 25:2593–602. doi: 10.1111/hex.13592, PMID: 35999687 PMC9615057

[53] Eitan T Gazit T . Leader behaviors in Facebook support groups: an exploratory study. Curr Psychol. (2023) 42:9691–707. doi: 10.1007/s12144-021-02262-w

[54] Mansour A . Shared information practices on Facebook: the formation and development of a sustainable online community. J Documentation. (2020) 76:625–46. doi: 10.1108/JD-10-2018-0160

[55] Roth-Cohen O . Viral feminism: MeToo networked expressions in feminist Facebook groups. Feminist Media Stud. (2022) 22:1695–711. doi: 10.1080/14680777.2021.1906295

[56] Frey E Bonfiglioli C Brunner M Frawley J . Parents' use of social media as a health information source for their children: a scoping review. Acad Pediatr. (2022) 22:526–39. doi: 10.1016/j.acap.2021.12.006, PMID: 34906742

[57] Seymour-Smith M Cruwys T Haslam SA Brodribb W . Loss of group memberships predict depression in postpartum mothers. Soc Psychiatry Psychiatr Epidemiol. (2017) 52:201–10. doi: 10.1007/s00127-016-1315-3, PMID: 27896374

[58] Schillinger D Chittamuru D Ramírez AS . From 'infodemics' to health promotion: a novel framework for the role of social media in public health. Am J Public Health. (2020) 110:1393–6. doi: 10.2105/AJPH.2020.305746, PMID: 32552021 PMC7427212

[59] Huo J Desai R Hong YR Turner K Mainous AG III Bian J . Use of social media in health communication: findings from the health information national trends survey 2013, 2014, and 2017. Cancer Control. (2019) 26:1073274819841442. doi: 10.1177/1073274819841442, PMID: 30995864 PMC6475857

[60] Ghahramani A de Courten M Prokofieva M . The potential of social media in health promotion beyond creating awareness: an integrative review. BMC Public Health. (2022) 22:1–13. doi: 10.1186/s12889-022-14885-0, PMID: 36544121 PMC9770563

[61] McNamara N Stevenson C Costa S Bowe M Wakefield J Kellezi B . Community identification, social support, and loneliness: the benefits of social identification for personal well-being. Br J Soc Psychol. (2021) 60:1379–402. doi: 10.1111/bjso.12456, PMID: 33942319 PMC8518584

[62] Gazit T Mass H Bronstein J . Examining Facebook groups engaging in reading experiences: the interactive therapeutic process perspective. Empirical Stud Arts. (2023) 41:259–83. doi: 10.1177/02762374221118522

[63] Seekis V Bradley GL Duffy AL . Does a Facebook-enhanced Mindful Self-Compassion intervention improve body image? An evaluation study. Body Image. (2020) 34:259–69. doi: 10.1016/j.bodyim.2020.07.006, PMID: 32717627

[64] Prescott J Rathbone AL Brown G . Online peer to peer support: qualitative analysis of UK and US open mental health Facebook groups. Digital Health. (2020) 6:2055207620979209. doi: 10.1177/2055207620979209, PMID: 33354335 PMC7734541

[65] Cauberghe V Van Wesenbeeck I De Jans S Hudders L Ponnet K . How adolescents use social media to cope with feelings of loneliness and anxiety during COVID-19 lockdown. Cyberpsychology Behavior Soc Networking. (2021) 24:250–7. doi: 10.1089/cyber.2020.0478, PMID: 33185488

[66] Thomas L Orme E Kerrigan F . Student loneliness: the role of social media through life transitions. Comput Educ. (2020) 146:103754. doi: 10.1016/j.compedu.2019.103754

[67] Gao J Zheng P Jia Y Chen H Mao Y Chen S . Mental health problems and social media exposure during COVID-19 outbreak. PloS One. (2020) 15:1–10. doi: 10.1371/journal.pone.0231924, PMID: 32298385 PMC7162477

[68] Geirdal A.Ø. Ruffolo M Leung J Thygesen H Price D Bonsaksen T . Mental health, quality of life, wellbeing, loneliness and use of social media in a time of social distancing during the COVID-19 outbreak: a cross-country comparative study. J Ment Health. (2021) 30:148–55. doi: 10.1080/09638237.2021.1875413, PMID: 33689546

[69] Helm PJ Jimenez T Galgali MS Edwards ME Vail KE Arndt J . Divergent effects of social media use on meaning in life via loneliness and existential isolation during the coronavirus pandemic. J Soc Pers Relat. (2022) 39:1768–93. doi: 10.1177/02654075211066922, PMID: 35664681 PMC9096014

[70] Ya'ari K Yeshua-Katz D Segal Engelchin D . The dark side of online support groups: conflictual experiences in single-child parents' Facebook groups. Soc Media + Soc. (2023) 9:20563051231179697. doi: 10.1177/20563051231179697

[71] Gal L Bronstein J Gazit T . Navigating parenthood in the digital landscape: a study on Facebook parenting community administrators. J Documentation. (2025) 81:638–56. doi: 10.1108/JD-09-2024-0233

[72] Kelly KJ Doucet S Luke A Azar R Montelpare W . Exploring the use of a Facebook-based support group for caregivers of children and youth with complex care needs: qualitative descriptive study. JMIR Pediatr Parenting. (2022) 5:e33170. doi: 10.2196/33170, PMID: 35671082 PMC9214619

[73] Berger MN Taba M Marino JL Lim MS Cooper SC Lewis L . Corrigendum to: social media's role in support networks among LGBTQ adolescents: a qualitative study. Sexual Health. (2021) 18:421–31. doi: 10.1071/SH21110, PMID: 34706814

[74] Godard R Holtzman S Duffield EM Do E Chong G Mathieson C . Stuff that only mixed-race people would understand': community and identity-related experiences in online groups for multiracial people. Asian J Soc Psychol. (2024) 27:672–85. doi: 10.1111/ajsp.12623

[75] Rose L Novice M Kobayashi S Minta A Novice T Sicco KL . Characterization of the role of Facebook groups for patients who use scalp cooling therapy: a survey study. Supportive Care Cancer. (2024) 32:351. doi: 10.1007/s00520-024-08534-y, PMID: 38748328 PMC11096238

[76] Gazit T . Key motivations for leading Facebook communities: a uses and gratifications approach. Aslib J Inf Manage. (2021) 73:454–72. doi: 10.1108/AJIM-11-2020-0379

[77] Gazit T Bronstein J Amichai-Hamburger Y Aharony N Bar-Ilan J Perez O . Active participants and lurkers in online discussion groups: an exploratory analysis of focus group interviews and observation. Inf Res. (2018) 23.

[78] Amichai-Hamburger Y Gazit T Bar-Ilan J Perez O Aharony N Bronstein J . Psychological factors behind the lack of participation in online discussions. Comput Hum Behav. (2016) 55:268–77. doi: 10.1016/j.chb.2015.09.009

[79] Naveh S Bronstein J . Sense making in complex health situations. Aslib J Inf Manage. (2019) 71:789–805. doi: 10.1108/AJIM-02-2019-0049

[80] Rosenberg H Kohn A . Temptations of fluency and dilemmas of self-definition: Stutterers’ usage and avoidance of new media technologies. Comput Hum Behav. (2016) 62:536–44. doi: 10.1016/j.chb.2016.04.008

[81] Nordberg OE Wake JD Nordby ES Flobak E Nordgreen T Mukhiya SK . Designing chatbots for guiding online peer support conversations for adults with ADHD. In: International workshop on chatbot research and design. Springer International Publishing, Cham (2019). p. 113–26.

[82] Young J Jawara LM Nguyen DN Daly B Huh-Yoo J Razi A . "The role of AI in peer support for young people: A study of preferences for human-and AI-generated responses". In: Proceedings of the 2024 CHI conference on human factors in computing systems. Honolulu Hawai’i (2024). p. 1–18.

